# Supplementary material for: Splice-Junction-Based Mapping of Alternative Isoforms in the Human Proteome
Source: Cell Rep. Author manuscript; Available in PMC 2020 Jan 15. (PMC6961840; doi:10.1016/j.celrep.2019.11.026)
Supplement: 2 [file NIHMS1546469-supplement-2.zip › DF1/PXD010154/Testis/Testis_6_SVIL_RPELCTSHSETPTVDDEEKVDER.pdf]

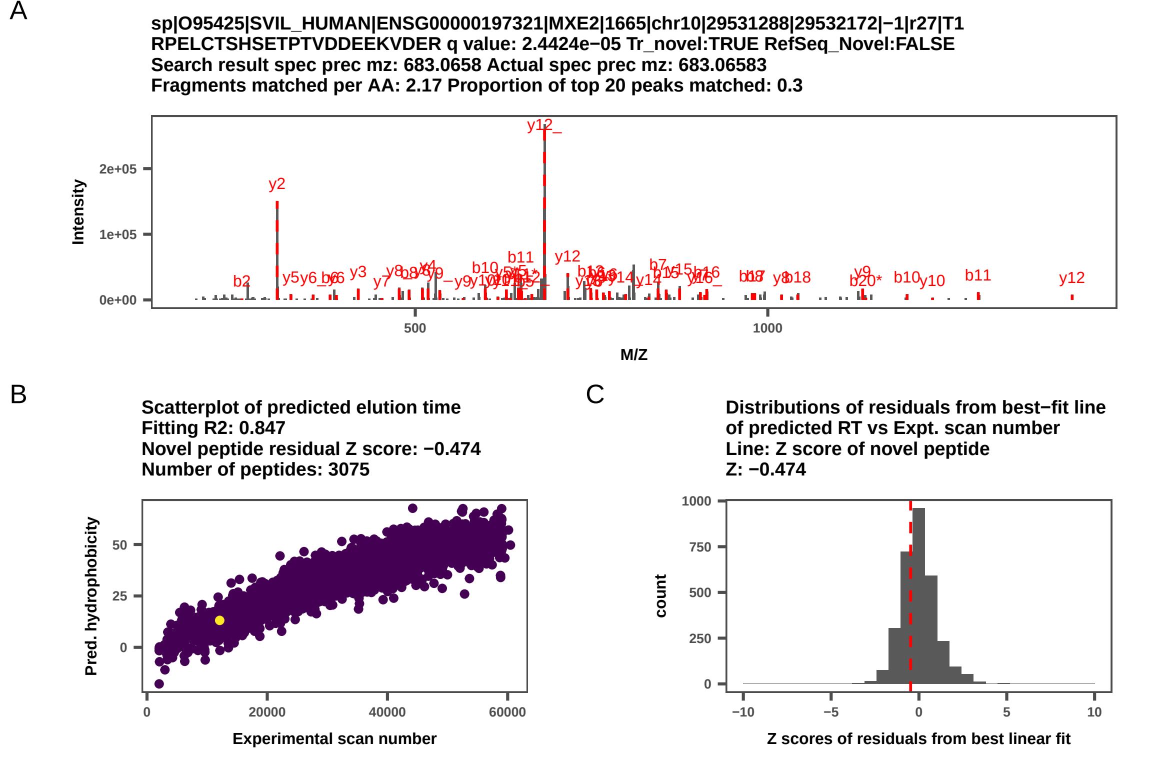
A
sp|O95425|SVIL_HUMAN|ENSG00000197321|MXE2|1665|chr10|29531288|29532172|−1|r27|T1
RPELCTSHSETPTVDDEEKVDER q value: 2.4424e−05 Tr_novel:TRUE RefSeq_Novel:FALSE
Search result spec prec mz: 683.0658 Actual spec prec mz: 683.06583
Fragments matched per AA: 2.17 Proportion of top 20 peaks matched: 0.3
Intensity
2e+05
1e+05
0e+00
500
1000
M/Z
y12_
y2
b2
y3
y7
b11
y12
b7
b10
y10
b11
y12
B
Scatterplot of predicted elution time
Fitting R2: 0.847
Novel peptide residual Z score: −0.474
Number of peptides: 3075
Pred. hydrophobicity
50
25
0
0
20000
40000
60000
Experimental scan number
C
Distributions of residuals from best−fit line
of predicted RT vs Expt. scan number
Line: Z score of novel peptide
Z: −0.474
count
1000
750
500
250
0
−10
−5
0
5
10
Z scores of residuals from best linear fit
